# Criticism and Depression among the Caregivers of At-Risk Mental State and First-Episode Psychosis Patients

**DOI:** 10.1371/journal.pone.0149875

**Published:** 2016-02-26

**Authors:** Yumiko Hamaie, Noriyuki Ohmuro, Masahiro Katsura, Chika Obara, Tatsuo Kikuchi, Fumiaki Ito, Tetsuo Miyakoshi, Hiroo Matsuoka, Kazunori Matsumoto

**Affiliations:** 1 Department of Psychiatry, Tohoku University Hospital, Sendai, Miyagi, Japan; 2 Department of Psychiatry, Tohoku University Graduate School of Medicine, Sendai, Miyagi, Japan; 3 Chiba Prison, Chiba-shi, Chiba, Japan; 4 Department of Preventive Psychiatry, Tohoku University Graduate School of Medicine, Sendai, Miyagi, Japan; Maastricht University, NETHERLANDS

## Abstract

Expressed emotion (EE), especially criticism, is an important predictor of outcomes for the patient for a wide range of mental health problems. To understand complex links between EE and various relevant variables in early phase psychosis, this study examined criticism, distress of caregivers, other patients’, and caregivers’ variables, and links between criticism and these variables in those with at-risk mental state (ARMS) for psychosis and first-episode psychosis (FEP). The participants were 56 patients (mean age 18.8 ± 4.2 years) with ARMS and their caregivers (49.4 ± 5.8 years) and 43 patients (21.7 ± 5.2 years) with FEP and their caregivers (49.3 ± 7.4 years). We investigated criticisms made by caregivers using the Japanese version of the Family Attitude Scale and caregiver depressive symptoms via the self-report Beck Depression Inventory. We also assessed psychiatric symptoms and functioning of the patients. Approximately one-third of caregivers of patients with ARMS or FEP had depressive symptoms, predominately with mild-to-moderate symptom levels, whereas only a small portion exhibited high criticism. The level of criticism and depression were comparable between ARMS and FEP caregivers. The link between criticism, caregivers’ depression, and patients’ symptoms were observed in FEP but not in ARMS caregivers. These findings imply that the interaction between criticism and caregivers’ and patients’ mental states may develop during or after the onset of established psychosis and interventions for the caregivers should be tailored to the patient’s specific stage of illness. Interventions for FEP caregivers should target their emotional distress and include education about patient’s general symptoms.

## Introduction

The family environment may alter both the onset [1] and outcome [2, 3] of psychosis and thus research investigating the family environment in early phase psychosis, that is, those with at-risk mental state (ARMS) and first-episode psychosis (FEP) [4–9], has important clinical implications. Although clinical guidelines recommend family interventions for ARMS and FEP [10], the nature of the family environment in early phase psychosis appears different from that of later or chronic phases [11, 12]. Conventional family interventions for families of people with early psychosis are not as effective as for families of chronic patients [13–15]. Therefore, to optimize family interventions in the early phase of illness it is necessary to gain further understanding of the family environment in early psychosis, including ARMS and FEP.

Research focusing on the family environment of people with mental illness has examined the clinical influence and underlying mechanisms of a family’s expressed emotion (EE). EE generally consists of criticism, hostility, and emotional over-involvement, and is an important predictor of outcome for a wide range of mental health problems [12, 16, 17]. Although previous studies examined the relationship between EE and symptoms and functioning of patients [18, 19], many did not differentiate the components of EE. However, critical components (i.e., criticism and hostility) and emotional over-involvement are known to be functionally different, at least in the early phase of mental illness [11, 20–22]. Among the three components of EE, criticism is regarded as the principal component [17] and impacts negatively the course of psychotic illness. Receiving criticism from family members in the early stages of schizophrenia is known to be associated with the number of relapses and the severity positive symptoms over time [23], and family criticism in patients with ARMS predicts an increase in positive symptoms over time [7]. Thus, it seems reasonable to investigate separately the link between criticism and relevant variables in the early phase of mental illness, to prevent the negative effect of criticism in this early stage.

Among such variables, distress or burden of families [2, 4, 24] appear important in the early course of psychosis. Family members who care for people with mental illness experience psychological distress, including depression, anxiety, feelings of loss, and embarrassment [25–27]. Some family members may also show psychiatric symptoms equivalent to psychiatric disorders themselves [4, 25, 28–30] and the distress of families might be stronger earlier in the course of psychosis [31]. Previous studies have demonstrated that the distress of family members of individuals with schizophrenia is linked to EE [2, 25, 30], and several studies have showed that emotional over-involvement, but not criticism, is linked to distress within the family of those with FEP [11, 21, 32]. In contrast, one study recently demonstrated that criticism is also linked to the burden of families of those of early psychosis within 3 years of FEP [33]. Therefore, it remains inconclusive as to whether criticism is associated with the distress or burden of families of FEP. Further, research on the family environment of individuals with ARMS is in its infancy. Thus, despite the importance of families in the care of patients with ARMS, no studies have examined possible links between criticism and distress of caregivers of people with ARMS.

Another important variable that could affect criticism is patients’ symptoms. Although one might expect families to be more critical when patients are more symptomatic, findings across studies of FEP and ARMS are inconsistent. Some studies have found associations between criticism and symptoms of patients with FEP [9, 34, 35], but others did not [11]. Similarly, although one study found an association between criticism and symptoms of patients with ARMS [9], another did not [36].

To understand the complex links between EE and various relevant variables, and differences in the family environment between ARMS and FEP, this study examined criticism, distress of caregivers, other patients’, and caregivers’ variables, and links between criticism and these variables in FEP and ARMS. In particular, we were interested in whether the depression of family members and symptoms of patients were associated with family criticism toward patients with ARMS or FEP, and whether these associations differed between ARMS and FEP families.

## Materials and Methods

### Sample

Patients with ARMS and FEP were recruited through the Sendai ARMS and first episode (SAFE) clinic, which is a specialized clinic in the Department of Psychiatry, Tohoku University Hospital in Sendai, Japan [37, 38]. All participants were recruited by a psychiatrist of the SAFE clinic and were required to be Japanese speaking and between the ages of 15 and 35 years old. General exclusion criteria included any history of neurological disorder, head trauma with loss of consciousness, mental retardation by the Diagnostic and Statistical Manual of Mental Disorders IV Text Revision (DSM-IV-TR) criteria [39], or substance abuse or addiction within 1 year of recruitment. This study was conducted according to the principles of the Declaration of Helsinki and approved by the Ethics Committee of Tohoku University Graduate School of Medicine and Tohoku University Hospital. All caregivers and patients provided written informed consent. If patients were under 18 years of age, parents provided written informed consent and the participant gave written assent.

#### ARMS cohort

Patients with ARMS met one or more of the following ultra-high risk criteria [40, 41] for: (i) attenuated psychotic symptoms; (ii) brief, limited intermittent psychotic symptoms; or, (iii) state and trait risk factors (a recent decline in functioning, plus a first-degree relative with either psychosis or a schizotypal personality disorder). Attenuated psychotic symptoms were assessed using a previously validated Japanese version [42] of the Comprehensive Assessment of ARMS (CAARMS) [43], a semi-structured interview tool designed to evaluate psychopathology and determine whether the ultra-high risk criteria are met [42]. Exclusion criteria for ARMS were (i) history of previous psychotic disorder or manic episodes that fulfilled the diagnostic criteria of bipolar I disorder specified in the DSM-IV-TR [39]; (ii) patients with a serious risk of suicide or violence due to personality disorder.

#### FEP cohort

Individuals with FEP met the CAARMS-J criteria for psychosis [44]. In addition, each had a Positive and Negative Syndrome Scale [45] score of 4 or more, with positive scale items of delusion, hallucinatory behavior, grandiosity, or suspiciousness, or general scale items of unusual thought content for more than 1 week.

#### Caregivers

Primary caregivers were defined as close relatives responsible for the care of the individuals with ARMS or FEP, and included parents, spouses, and other relatives. If there was more than one caregiver, they were asked to determine who was the most involved in providing care to the patients.

### Participation

Participants were consecutively recruited between July 2008 and March 2015. During the period of study, 151 patients (78 ARMS and 73 FEP) met the eligibility criteria. Sixteen patients (8 ARMS and 8 FEP) did not have proper caregivers nearby. Among the caregivers of the remaining 135 patients, those of 99 (73%) patients (56 ARMS and 43 FEP) consented to participate in this study.

### Assessments

Both patients and their caregivers were assessed within 1 month of intake by one of psychiatrists of the SAFE clinic who had sufficient experience of seeing patients with ARMS and FEP, and who had been trained as a clinical researcher more than 3 years, and was familiar with all of the instruments used in this study.

#### Assessment of patients

Psychopathological symptoms associated with psychotic illness were assessed using the Positive and Negative Syndrome Scale (PANSS, [45]), a 30-item scale with three symptom subscales: positive, negative, and general. The extensively used Japanese version of PANSS was translated and applied by Yamada et al. [46, 47]. Global functioning was assessed using the Global Assessment of Functioning (GAF) scale, and the current level of social and occupational functioning was assessed using the Social and Occupational Functioning Assessment Scale (SOFAS), which is included in the DSM-IV-TR[39].

#### Assessment of caregivers

Criticism was assessed by means of the Family Attitude Scale (FAS, [48, 49]), which was validated in a Japanese sample by Fujita et al. [48]. The FAS is a self-report measure of critical comments by family members, consisting of 30 questions (e.g., ‘I wish he were not here’; ‘He is a real burden’; ‘I find myself saying nasty or sarcastic things to him’), using a Likert scale rating from 0 (never) to 4 (everyday). Thus, FAS scores are in the range 0–120, with higher scores indicating higher levels of criticism. Compared with the Camberwell Family Interview (CFI), a standard interview method for EE assessment, the sensitivity and specificity of the Japanese FAS to detect high criticism were 100% and 88.5%, respectively, using a cut-off point of 60 [48]. This cut-off was used for the present study.

Caregiver depressive symptoms were assessed through the 21-item self-report Beck Depression Inventory, 2nd edition (BDI-II, [50]), with scores in the range 0–63. Severity of depression was categorized into 4 levels: normal to minimal (0–13), mild (14–19), moderate (20–28), and severe (29–63) [51]. The reliability and validity of the Japanese version of the BDI-II has been established by Kojima et al. [52].

### Statistical analysis

For continuous variables, the statistical significance of differences between groups was assessed with Student *t*-tests if variances were normally distributed, and otherwise with Mann-Whitney *U-*tests. Categorical variables were analyzed using chi-square tests. Bivariate correlations were performed to examine associations of the FAS score with the BDI-II, PANSS, GAF, and SOFAS scores, and with demographic data. To determine which variables best predicted the FAS score, a series of stepwise regression analyses were performed. Statistical differences were determined using two-tailed tests and a significance level of *p* < 0.05. All statistical analyses were performed using the Statistical Package for the Social Sciences (SPSS) 20.0 for Windows (SPSS Inc., Chicago, Illinois).

## Results

### Sample characteristics

The demographic and clinical data of patients are presented in Table 1. Both patient groups were predominantly female (ARMS 68% vs. FEP 67%). Those with ARMS were younger and had a lower educational level than patients with FEP. Of the 56 patients with ARMS, 86% (*n* = 48) were classified into the attenuated psychotic symptoms group, 4% (*n* = 2) were classified into the brief limited intermittent psychotic symptoms group, 9% (*n* = 5) were classified into a group of both attenuated psychotic symptoms and state and trait risk factors, and 2% (*n* = 1) were classified into a group of both attenuated psychotic symptoms and brief limited intermittent psychotic symptoms. The FEP group consisted of schizophrenia (*n* = 28), schizophreniform disorder (*n* = 4), bipolar disorder with psychotic features (*n* = 3), delusional disorder (*n* = 1), brief psychotic disorder (*n* = 1), and psychosis not otherwise specified (*n* = 6) according to the DSM-IV-TR [39]).

**Table 1:** Baseline Characteristics of Patients with At-risk Mental State (ARMS) and First-episode Psychosis (FEP).

Characteristic	ARMS	FEP	Statistic	*p*
	(*n* = 56)	(*n* = 43)		
Age (mean ± SD, years)	18.8 ± 4.2	21.7 ± 5.2	*U* = 1671	.001
Gender (male/female)	18/38	14/29	*χ*^*2*^ = 0.00	.97
Education (mean ± SD, years)	11.8 ± 2.3	12.7 ± 1.9	*U* = 1513	.03
Living with caregivers (%)	55 (98.2%)	35 (81.4%)	*χ*^*2*^ = 8.33	.009
Occupation (%)			*χ*^*2*^ = 6.38	.10
Student	45 (80.4%)	26 (60.5%)		
Employed	3 (5.4%)	8 (18.6%)		
Housewife	2 (3.6%)	1 (2.3%)		
Unemployed	6 (10.7%)	8 (18.6%)		
Outpatient/Inpatient	54/2	27/16	*χ*^*2*^ = 18.50	< .001
PANSS^a^ (mean ± SD)				
Total	57.3 ± 13.1	75.7 ± 19.3	*t* = 5.28	< .001
Positive	13.0 ± 2.9	18.8 ± 4.8	*t* = 6.89	< .001
Negative	12.4 ± 4.9	17.7 ± 6.8	*U* = 1682	< .001
General	32.0 ± 6.9	39.2 ± 10.5	*U* = 1649	< .001
GAF^b^ (mean ± SD)	49.4 ± 7.1	40.6 ± 10.4	*U* = 588	< .001
SOFAS^c^ (mean ± SD)	51.3 ± 8.3	43.8 ± 11.5	*U* = 672	< .001

^a^Positive and Negative Syndrome Scale (PANSS).

^b^Global Assessment of Functioning (GAF).

^c^Social and Occupational Functioning Assessment Scale (SOFAS).

More patients with ARMS lived with their caregivers than patients with FEP, and most patients were students or employed. Total PANSS scores as well as positive symptoms, negative symptoms, general psychopathology, and global and social functioning were worse in individuals with FEP than those with ARMS.

The demographic data of caregivers is presented in Table 2. The study enrolled 56 caregivers of patients with ARMS and 43 caregivers of patients with FEP. There were no differences in age and educational level between these groups, but mothers were more likely to be the primary caregivers than fathers (ARMS 75% vs. FEP 81%).

**Table 2:** Baseline Characteristics of Caregivers of Patients with At-risk Mental State (ARMS) and First-episode Psychosis (FEP).

Characteristic	ARMS	FEP	Statistic	*p*
	(*n* = 56)	(*n* = 43)		
Age (mean ± SD, years)	49.4 ± 5.8	49.3 ± 7.4	*U* = 1272	.63
Relationship			*χ*^*2*^ = 6.30	.10
Father (%)	14 (25.0%)	5 (11.6%)		
Mother (%)	42 (75.0%)	35 (81.4%)		
Sibling (%)	0 (0%)	1 (2.3%)		
Spouse (%)	0 (0%)	2 (4.7%)		
Education (mean ± SD, years)	13.4 ± 1.9	13.0 ± 1.8	*U* = 977	.56
FAS^a^ (mean ± SD)	31.2 ± 19.5	26.9 ± 16.8	*t* = 1.15	.25
High criticism (%)	3 (5.3%)	2 (4.7%)	*χ*^*2*^ = 0.03	.87
Low criticism (%)	53 (94.6%)	41 (95.3%)		
BDI-II^b^ (mean ± SD)	11.4 ± 8.5^c^	11.9 ± 9.0^d^	*U* = 1139	.93
Severe (%)	1 (1.8%)	3 (7.3%)		
Moderate (%)	11 (20.0%)	2 (4.9%)		
Mild (%)	6 (10.9%)	8 (19.5%)		
None (%)	37 (67.3%)	28 (68.3%)		

^a^Family Attitude Scale (FAS).

^b^Beck Depression Inventory II (BDI-II).

^c^Data missing for 1 participant.

^d^Data missing for 2 participants.

### FAS and BDI-II scores of caregivers

Caregivers of ARMS and FEP patients differed in neither the mean FAS scores nor the proportion given high criticism. The mean BDI-II scores were comparable; the proportion of mild-to-moderate depression [51] was 30.3% (n = 17) in caregivers of ARMS patients and 23.8% (n = 10) in caregivers of FEP patients. However, only 1 (1.8%) and 3 (7.3%) caregivers of ARMS and FEP patients, respectively, presented severe depressive symptomatology.

### Relationship between FAS and variables in caregivers and patients

Criticism of FEP caregivers was correlated with depression of the caregivers and patients’ negative and general psychopathology symptoms (Table 3). In contrast, the criticism of ARMS caregivers was only correlated with the educational levels of caregivers.

**Table 3:** Correlations Between FAS Scores and Patients’ Clinical and Functional Variables, Caregivers’ Depressive Symptoms, and Demographic Variables.

Variable	FAS scores of ARMS caregivers		FAS scores of FEP caregivers	
	*r*	*p*	*r*	*p*
Patient variables				
PANSS				
Total	0.11	.42	*0*.*25*	.*12*
Positive	0.13	.34	0.13	.43
Negative	*-0*.*03*	.*86*	0.35	.02*
General	*0*.*10*	.*47*	0.40	.009**
GAF	*-0*.*04*	.*78*	*-0*.*28*	.*07*
SOFAS	*-0*.*07*	.*63*	*-0*.*27*	.*09*
Age	*0*.*21*	.*12*	*-0*.*03*	.*83*
Education	*0*.*12*	.*40*	0.13	.40
Caregiver variables				
BDI-II	0.18	.18	*0*.*57*	*<* .001**
Age	0.02	.87	*-0*.*12*	.*47*
Education	*0*.*45*	.001**	*-0*.*07*	.*68*

Spearman’s rho correlation coefficients are in italics. Pearson’s product moment correlation coefficients are in normal typeface.

* *p* < .05,

**p < .01.

The comparison of correlation coefficients between ARMS and FEP groups (Table 4) revealed that the correlation between criticism and depression of the caregiver was significantly larger in the FEP than the ARMS group. Although the correlations between criticism and patients’ symptoms did not differ between the two groups, those between criticism and the patients’ negative symptoms approached significance, with a tendency toward larger correlations in the FEP than the ARMS group. The correlation between criticism and the educational level of the caregivers was significantly larger in the ARMS than the FEP group.

**Table 4:** Comparison of Correlations Between ARMS and FEP Groups.

Variable	Test for equality of correlation coefficients	
	*z*	*p*
Patient variables		
PANSS		
Total	0.68	.49
ositive	0.03	.98
Negative	1.88	.06
General	1.57	.12
GAF	1.20	.23
SOFAS	0.99	.32
Age	1.17	.24
Education	0.07	.94
Caregiver variables		
BDI-II	2.19	.03*
Age	0.66	.51
Education	2.64	.008**

* *p* < .05,

**p < .01.

Regression analysis revealed that in the ARMS group FAS scores were only explained by the educational level of the caregiver (adjusted R^2^ = 0.19, multiple R = 0.46, F (1, 44) = 11.58, *p* = .001), which made a significant independent contribution when all variables were considered simultaneously (β = 0.46, B = 4.98, *p* = .001). In the FEP group, FAS scores were explained by the BDI-II scores of caregivers and the general psychopathology scores of PANSS (adjusted R^2^ = 0.46, multiple R = 0.70, F (2, 30) = 14.39, *p <* .*001*). Both made significant independent contributions when all of the variables were considered simultaneously (depressive symptoms of caregiver: β = 0.47, B = 0.96, *p* = .001; general psychopathology score: β = 0.42, B = 0.71, *p* = .004).

## Discussion

The present study examined family environmental variables that may be linked with criticism of patients by caregivers and found that links between criticism, patients’ symptoms, and caregivers’ depression were observed in FEP caregivers but not in ARMS caregivers.

The finding that criticism by caregivers was associated with their depressive symptoms in the FEP group is consistent with earlier research that uncovered correlations between criticism and depression, and anxiety, in caregivers of individuals with early psychosis who were within 3 years of first psychosis episode [33]. In contrast, criticism by ARMS caregivers was not associated with their depressive symptoms. Although the level of criticism and depression was comparable among ARMS and FEP caregivers, the link of criticism with caregiver depression was only observed in the FEP caregivers. This suggests that the interaction between the emotional distress of caregivers and criticism might only be observed after the onset of psychosis, i.e., not at the at-risk stage. Generally, individuals with FEP present more severe symptoms than those with ARMS, and FEP caregivers have typically been involved in a patient’s illness longer than ARMS caregivers. Thus, the interaction between criticism and caregiver distress may develop during the course of the illness. This notion is consistent with the repeatedly replicated finding of an association between criticism and duration of untreated psychosis in patients with FEP [11, 53]. EE was hypothesized to be an adaptive reaction to perceived loss [53] or a coping strategy to reduce the perceived stressfulness of the caring role [18]. Therefore, the present finding of an association between criticism and depression in the FEP families implies that interventions to reduce the burden and distress of the family may be effective to prevent maladaptive reactions or coping of families, and thus reduce the risk of increasing criticism of patients with early psychosis by their families.

In the present study, criticism was associated with patients’ general symptoms in FEP caregivers. Although there was an association between criticism and patients’ negative symptoms in the correlation analysis, the association was not present in the regression analysis. The association between families’ criticism and general symptoms other than positive and negative symptoms of FEP patients is consistent with previous studies [9, 33]. According to the attribution model of EE [3, 54], criticism would be associated with caregivers’ attribution style. Therefore, FEP caregivers may view general symptoms such as anxiety and behavioral problems as more controllable and reactive factors, rather than being indicative of an underlying illness [55]. In contrast to FEP caregivers, criticism was not associated with any symptoms in ARMS patients. This suggests that the interaction between criticism and symptoms of patients develops after psychosis is established. However, the finding contradicts a previous study [9] that found associations of criticism with positive, negative, and general symptoms of patients in a mixed sample of ARMS and FEP, with no group differences, suggesting that criticism and patients’ symptoms might interact even during the at-risk stage. The partially inconsistent findings between the present and the previous study [9] may lie in the different symptom characteristics of patients with ARMS and FEP. In the previous study, in contrast to the present study, there was no difference in positive and negative symptoms between ARMS and FEP groups, and general symptoms were more severe in ARMS than FEP [9]. Differences in symptom characteristics could reasonably affect the interaction of criticism with patients’ symptoms.

Contrary to our expectation, the education level of ARMS caregivers was moderately associated with criticism. Although the precise reason is unclear, we speculate that parents with a high educational level have high expectations of their child’s ability to control his or her illness and behavior; thus the parents become more critical [20–22, 56]. More research is necessary to determine what factors mediate this relationship.

Several methodological limitations are apparent in this study. First, the sample size was relatively small and the number of caregivers was also small, which may restrict the generalizability of the findings. Second, we only measured criticism using self-report ratings and did not rate other EE components such as emotional over-involvement and hostility. Therefore, the results should be replicated using comprehensive interview-based ratings (e.g., the CFI). Finally, the cross-sectional study design limits extrapolation of the findings beyond the time of the investigation. A longitudinal follow-up study should examine whether criticism and distress among caregivers can predict outcomes among patients with FEP and ARMS.

## Conclusions

According to the present findings, family interventions for FEP should focus preferentially on the understanding of, and efforts to alleviate, the distress and burden of families. In addition, because ARMS caregivers had depressive symptoms equivalent to FEP caregivers, and the link between criticism and family depression had been established before the initial treatment of FEP, interventions should be also provided for caregivers of patients with ARMS. Moreover, education that promotes the understanding of caregivers regarding general symptoms and negative symptoms of patients should be more important for FEP caregivers. Interventions for the caregivers should be tailored to the patient’s specific stage of illness and should target the emotional distress of caregivers. Moreover, interventions should aim to prevent exacerbation of the criticism of patients by their caregivers during early psychosis. Such an approach would reduce the burden of disease for both the patient and caregiver.

## Supporting Information

S1 Materials Data File in this study. (PDF)
