# Correction: Criticism and Depression among the Caregivers of At-Risk Mental State and First-Episode Psychosis Patients

**DOI:** 10.1371/journal.pone.0156590

**Published:** 2016-05-27

**Authors:** Yumiko Hamaie, Noriyuki Ohmuro, Masahiro Katsura, Chika Obara, Tatsuo Kikuchi, Fumiaki Ito, Tetsuo Miyakoshi, Hiroo Matsuoka, Kazunori Matsumoto

In the Funding section, the first grant number from the Japan Society for the Promotion of Science is listed incorrectly. The correct number for the first grant is: Grant-in-Aid for Young Scientist (B) 25780404.
