# Supplementary material for: Criticism and Depression among the Caregivers of At-Risk Mental State and First-Episode Psychosis Patients
Source: PLoS One. 2016 Feb 26;11(2):e0149875. doi: 10.1371/journal.pone.0149875 (PMC4769225; doi:10.1371/journal.pone.0149875)
Supplement: S1 Materials — (PDF) [file pone.0149875.s001.pdf]

group	sex	age	education	age of caregiver	education of caregiver	FAS	BDI-II
arms	f	20	13.5	48	14	13	8
arms	f	16	9.5	43	12	24	1
arms	m	20	13.5	48	14	73	4
arms	f	16	10.5	54	16	46	13
arms	f	17	10.5	50	16	17	20
arms	f	22	15.5	49	12	42	9
arms	f	17	10.5	45	16	45	9
arms	m	23	16	54	12	11	8
arms	m	23	16	55	16	53	22
arms	f	16	9.5	40	14	32	14
arms	m	15	9.5	45	12	23	21
arms	f	15	8.5	50	14	36	12
arms	f	16	10.5	43	16	35	12
arms	f	16	10.5	43	14	36	2
arms	f	16	10.5	47	14	23	10
arms	f	18	11.5	48	16	119	26
arms	m	16	10.5	48	16	9	0
arms	f	25	16	51	14	28	15
arms	f	16	10.5	49	16	35	22
arms	m	18	12.5	46	14	38	21
arms	f	18	12.5	47	14	40	10
arms	f	23	13	52	12	20	9
arms	f	17	11.5	41	12	18	18
arms	f	27	14	56	12	26	6
arms	m	22	12.5	50	12	38	8
arms	m	20	14.5	47	12	24	11
arms	f	32	16	62		56	6
arms	m	17	10.5	52	16	54	14
arms	f	15	9.5	55	12	5	1
arms	f	16	9.5	43	12	20	11
arms	m	18	12.5	54	16	29	7
arms	f	16	11.5	45	14	24	21
arms	f	22	9.5	60	13	62	19
arms	f	17	11.5	44	14	23	5
arms	f	16	11.5	46	12	32	20
arms	m	23	12	52	16	44	7
arms	m	21	15.5	57	16	16	1
arms	m	23	15.5	49		32	23
arms	f	15	8.5	53	12	37	23
arms	m	17	10.5	46	12	32	3
arms	m	13	6.5	55	12	2	0
arms	f	34	14	68	9	10	1
arms	f	14	8	54	12	22	
arms	f	26	16	51	12	25	8
arms	f	17	11.5	55	12	19	9
arms	m	16	11.5	57	16	51	6
arms	f	22	14.5	52	14	19	15
arms	m	18	12	45	12	18	24
arms	f	19	13.5	47	12	22	1
arms	f	15	9.5	38	12	13	43
arms	f	18	12	44	12	49	5
arms	m	16	12	45	12	12	11
arms	f	16	10.5	54	12	10	11
arms	f	18	11.5	49	14	29	13
arms	f	18	11.5	43	12	56	3
arms	f	17	11.5	42	12	18	4
fep	f	34	13	60	9	63	21
fep	f	19	15	53	14	29	11

fep	m	33	16	66	12	27	8
fep	f	19	12.5	47	12	17	10
fep	f	19	11.5	47	9	26	
fep	f	29	12	53		12	4
fep	f	18	12.5	54	12	3	
fep	f	19	12.5	44	14	85	18
fep	f	22	12	46	12	29	7
fep	f	15	9.5	51	16	32	11
fep	f	20	13.5	52	12	19	6
fep	f	16	9.5	37	12	6	6
fep	f	17	11.5	50	16	18	11
fep	f	17	10.5	46	16	28	10
fep	m	25	14	54	16	20	10
fep	f	21	14.5	47	14	15	6
fep	f	17	10.5	50	14	56	16
fep	f	20	13.5	52	12	40	11
fep	f	20	13	49	12	43	22
fep	m	23	16	50	14	37	6
fep	f	23	12	49		17	13
fep	f	18	11.5	50	16	18	0
fep	m	33	12	31		27	17
fep	m	26	12	53	14	17	6
fep	f	23	12	29		5	0
fep	f	17	11.5	43	14	47	17
fep	f	34	14	34		41	13
fep	f	27	16	60	12	18	7
fep	m	18	11.5	49	12	28	15
fep	f	23	16.5	63	13	48	7
fep	m	18	11.5	41	12	41	42
fep	f	28	9.5	57	12	12	5
fep	m	−1	12	50	12	11	36
fep	m	22	15	52	16	21	17
fep	m	29	16	59	14	17	8
fep	f	15	10	49	14	39	31
fep	m	16	9.5	47	12	10	14
fep	m	17	11.5	51	16	1	0
fep	f	20	13	50	14	16	6
fep	f	19	13.5	44		32	8
fep	m	19	13.5	53	14	24	2
fep	m	20	14.5	49	12	40	19
fep	m	19	13.5	49	12	21	11

PANSS total	PANSS positive	PANSS negative	PANSS general	GAF	SOFAS
89	19	24	46	35	40
64	14	17	33	50	50
68	15	7	46	45	45
117	20	37	60	30	35
67	13	19	35	55	55
63	13	11	39	45	45
45	10	9	26	55	55
55	10	13	32	45	45
53	14	9	30	55	60
59	13	13	33	40	40
65	12	18	35	45	45
60	15	12	33	50	55
49	9	8	32	50	50
66	18	15	33	60	75
48	11	10	27	53	53
54	12	10	32	55	60
70	16	13	41	50	50
60	14	12	34	55	55
42	10	8	24	55	70
52	13	11	28	51	55
45	9	11	25	45	45
52	11	10	31	38	38
49	12	8	29	45	45
60	12	16	32	45	45
44	10	8	26	65	65
52	14	9	29	55	55
70	17	17	36	45	45
58	14	14	30	45	45
56	17	10	29	50	50
45	12	7	26	55	55
65	14	15	36	55	60
59	15	12	32	40	40
43	9	9	25	55	55
57	11	17	29	50	50
59	11	11	37	45	45
43	8	8	27	60	60
71	18	17	36	50	50
48	10	12	26	50	50
51	10	12	29	50	50
42	13	9	20	45	45
73	15	15	43	55	55
72	17	17	38	45	55
62	15	13	34	55	55
47	11	11	25	43	43
57	14	13	30	40	40
63	14	13	36	40	50
66	18	13	35	55	65
50	9	8	33	45	45
49	12	9	28	50	50
57	16	13	28	57	57
33	7	10	16	65	65
48	11	7	30	51	62
48	12	8	28	45	45
60	13	13	34	45	45
51	11	10	30	60	60
99	25	21	53	25	25
96	22	25	49	31	35

70	16	20	34	40	40
76	15	25	36	41	41
67	19	10	38	51	51
77	25	14	38	25	25
70	26	10	34	45	45
122	23	34	65	25	25
73	15	16	42	35	35
52	12	11	29	50	50
93	19	22	52	35	35
82	21	20	41	45	50
56	14	8	34	45	45
103	25	20	58	30	40
43	14	8	21	55	75
61	18	12	31	50	60
91	22	21	48	45	45
72	20	16	36	35	35
105	27	32	46	30	30
66	17	10	39	50	50
50	13	8	29	55	55
63	21	14	28	35	45
				61	61
90	15	29	46	35	35
119	29	29	61	20	30
71	13	22	36	45	45
100	25	17	58	30	35
39	9	9	21	45	65
68	20	17	31	45	45
				30	40
80	17	18	45	45	60
55	15	7	33	45	45
90	21	23	46	45	
86	25	21	40	40	45
60	19	12	29	35	35
65	15	17	33	40	40
60	15	15	30	45	45
80	21	18	41	52	55
85	16	25	44	40	40
68	16	13	39	43	43
69	18	18	33	50	52
81	22	22	37	15	25
51	11	16	24	60	60
